# Cases in Practice

**Published:** 1882-06-20

**Authors:** A. A. Davidson

**Affiliations:** Milan, Tenn.


### CASES IN PRACTICE.

BY A. A. DAVIDSON, M. D., OF MILAN, TENN.

Having recently seen interesting items in the RECORD, on atropia, also on liquor ammoniæ, I send you the following cures which are at your disposal:

CASE I.—CONVULSIONS—In July, 1876, John Cunningham, aged 19 years, was attacked with violent convulsions from some trouble of the brain; they lasted him several weeks, and were so violent, at times, it would take four or five men to hold him still. Had nine physicians to see him during the time; all the remedies failed to have the desired effect, till I put him on large doses brom. potash, 40 to 90 grains at a dose, in conjunction with extract belladonna, $\frac{1}{4}$ to $\frac{1}{2}$ grain, which gave him immediate and *permanent* relief.

During his illness I concluded to try the antagonistic properties of opium and belladonna. I injected hypodermacally, 1-50 grain atropia, and in thirty minutes his pulse went up 30 beats; pupils dilated ; countenance assumed a distressed expression, skin purple, with spots and rashes on it, and he seemed to try to tear himself to pieces; scarcely could restrain him from violence; so much so that I became a little alarmed. I injected $\frac{1}{3}$ grain morphine, and in twenty minutes his pulse fell to ninety, a reduction of thirty beats, and he became as calm as before.

CASE II—INJURY TO THE EYE BY SPIRITS OF AMMONIA.—I see in the last number of the RECORD an account of a lady losing her eye by the explosion of an ammonia vial. About three months ago I received a box of drugs from Memphis, opened it and took out a bottle of aqua ammoniæ and put it on a shelf in my office, and it had a rubber cork in it, which excited the curiosity of my little boy, aged seven years. He took the bottle down, and the stopper flew out, and the liquid flew in his right eye and over his face. I was a few hundred yards from the house; was summoned to come to him in great haste—found him suffering intense pain, and bathing his eye with cold water, I opened it and found the conjunctiva very red and swollen. As I had been a practicing oculist for twenty years, I set about applying remedies. The first thing I did was to drop in a solution of sulph. morphia to give ease; next I used atropia, two grains to ounce, two or three times a day; kept him on this prescription for several days; also used cloths dipped in tea water, on the lid, alternated with mucilage of slippery elm bark.

The most severe and intense photophobia came on immediately, in *both* eyes, so much so that I was not able for three days to see their actual condition. His eye was very much swollen and exceedingly tender. On the fourth day he was able to open his eyes a little, from which time he recovered rapidly, with no bad results.

I think the *prompt* and active treatment saved his eye. His face was not damaged.

CASE III—LARGE DOSES FOWLER'S SOLUTION WITHOUT FATAL RESULTS —I had a case of typhoid fever, a young lady, a few years ago, and her father came to get some medicine for her sister, older than herself, for tertian ague, of long standing. I gave him a half ounce vial of Fowler's solution, with directions to give ten drops three times a day; also sent my typhoid case four ounce bottle turpentine emulsion, to be given in teaspoonful doses every two hours. Her father forgot the directions, and reversed the directions, and gave the Fowler's solution in *teaspoonful* doses every two hours till the young lady took three drachms in *four hours*. She soon became greatly prostrated, eyes swollen, tongue enlarged and protruded, and became paralyzed all over, so much so she could not raise hand or foot. The first thing she remembered or desired was *onions*; she ate some of them and soon recovered. She had no vomiting or purging, nor has she ever had any after unpleasant effects.

Mr. Editor, will you or any one explain her rapid recovery from so poisonous an agent, or did the onions cure her?

